# Books and Authors

**Published:** 1908-12

**Authors:** 


**A Treatise on the Principles and Practice of Gynecology.** By **E. C. Dudley,** A.M., M.D., Professor of Gynecology in the Northwestern University Medical School, Chicago. Fifth edition, thoroughly revised. Octavo, 806 pages, with 431 illustrations, of which 75 are in colors, and 20 full-page colored plates. (Cloth, $5.00 net; leather, $6.00 net; half-morocco, $6.50.) Lea & Febiger, Publishers, Philadelphia and New York, 1908.

Dudley's gynecology is too well-known to the medical profession to require a formal introduction at this time at our hands. It has met the approval of teachers, practitioners and students in the four preceding editions and, we cannot doubt, will receive even a warmer approbation in the fifth edition now issued. The general plan of the volume is the same as before, but a number of chapters have been changed to meet advances made of late. Two new chapters appear, one an introduction that comprises a portion of the author's presidential address before the American Gynecological Society at Niagara Falls, May, 1905; and the other, the final chapter, in incontinence of urine in women, also dealt with in that address. We made extended editorial comment on this address in the July, 1905, edition of the Journal, to which we invite the attention of our readers.

The illustrations are made from special drawings, all original work, and are particularly valuable to the practitioner. We are unacquainted with any similar treatise in which the illustrations are so profuse or so useful from the viewpoint of instruction. Dudley's gynecology will retain its place in the forefront among treatises on the diseases of women.

---

**Progressive Medicine,** Vol. No. 3, September, 1908. A Quarterly Digest of Advances, Discoveries and Improvements in the Medical and Surgical Sciences. Edited by **Hobart Amory Hare,** M.D., Professor of Therapeutics and Materia Medica in the Jefferson Medical College of Philadelphia. Octavo, 285 pages, with 30 engravings. Lea & Febiger, Publishers, Philadelphia and New York. (Per annum, in four cloth-bound volumes, $9.00; in paper binding, $6.00, carriage paid to any address.)

This number contains abstracts of medical progress under four general heads. Ewart, in the department of diseases of the thorax and its viscera, presents an excellent summary of recent literature pertaining to tuberculosis; also in regard to emphysema, as well as disturbances that arise in disorders of the heart, in blood pressure, in diseases of the lungs, and of the bloodvessels. Gottheil deals with dermatology and syphilis in splendid fashion, while Davis presents an unusually interesting synopsis of obstetric literature.

The branch relating to the nervous system is presented by William G. Spiller, covering everything of importance that has appeared relating to that topic.

Progressive medicine occupies a field apart from that of the magazine. It performs for the general practitioner, the surgeon and the specialist a most important service, bringing him knowledge which he could not otherwise obtain, either by his own efforts or in any other publication.

---

**Adenomyoma of the Uterus.** By **Thomas Stephen Cullen,** M.D., associate professor of gynecology in the Johns Hopkins University. Royal 8vo, pp. 270. Illustrated. Philadelphia and London: W. B. Saunders Co. (Cloth, $5.00.)

Whoever examines this work with care cannot fail to be impressed by several remarkable features that are developed in its pages. It is based on a study of about one hundred cases of adenomyoma which the author has encountered. This compound form of growth is less common than has been believed. Cullen informs us in this book that of 1,283 cases of myoma examined between April 1, 1893, and July 1, 1896, 5.7 per cent. were examples of adenomyoma. From his summary we extract data of importance.

He divides adenomyomata into three groups: (1) adenomyomata in which the uterus preserves a relatively normal contour; (2) subperitoneal or intraligamentary adenomyomata; (3) submucous adenomyomata. Lengthened menstrual periods, he says, are the first symptoms, the flow gradually assuming the proportions of hemorrhages. The pathology of this condition is dealt with in a thorough manner.

Cullen thinks the diagnosis clinically should not be difficult, especially of diffuse adenomyoma. His reasons are, (1) the bleeding is usually confined to the period; (2) there is usually much pain, referred to the uterus, at the period; (3) there is usually no intermenstrual discharge of any kind; (4) the uterine mucosa is perfectly normal and may be rather thick. No other pathological condition of the uterus, he affirms, as a rule, gives this clinical picture.

As to treatment, the only rational treatment is to remove the uterus. The patient's health, generally speaking, is being undermined by the hemorrhages and these can be controlled only by this procedure, a supravaginal hysterectomy without removal of the ovaries being advised. The entire book is full of interest and possesses great scientific value. It is handsomely printed in large type on heavy calendered paper. The engravings are superb specimens of art work,—author, artists and publishers having together made a most beantiful and instructive volume.

---

**Textbook of Operative Surgery,** covering the surgical anatomy and operative technic involved in the operations of general surgery designed for practitioners and students, by **Warren Stone Bickham,** M.D., Phar,M., junior surgeon to Touro Hospital, New Orleans. Third edition. 854 illustrations, pp. 1,206. W. B. Saunders Company, Philadelphia and London. 1908. (Price, cloth, $6.50 net.)

It has remained for Bickham to become one of the most famous of the younger authors on surgery. He has been fortunate, singularly so, in presenting the right kind of a surgical treatise and, also, in presenting it in the right way. The first edition, which appeared in 1903, received extended notice in the issue of the JOURNAL for January, 1904. At that time, among other things, we said the book was a distinct addition to the literature of surgery, and we may now add that it belongs to the permanent class of that literature.

In this edition, as in those preceding it, the latest operations are given with clearness and the best technic for their execution is described. We may cite Bickham's chapters on brain surgery and on the pancreas as instances of excellent detail and as examples of the very best modern scientific surgical instruction. The book abounds with evidence of thoughtful, studious work, is arranged in a manner that appeals to both student and practicing surgeon, and will prove of great value to him who must perforce practice surgery as well as medicine, and for which he must equip himself with literature which meets his necessities. Moreover, it will be needed by every teacher and instructor in surgery of whatever grade.

The changes in this edition are many. Twenty-nine pages have been omitted while one hundred and twenty-three have been added. Forty-two pictures have been omitted and forty-five have been redrawn, while three hundred and thirty-one have been added. The last edition contained nine hundred and eighty-four pages and five hundred and fifty-nine illustrations; there are in this one thousand and four pages and eight hundred and fifty-four illustrations. Bickham has made the best, far and away the best, operative surgery in the American language.

---

**The Practitioners' Visiting List** for 1909. An invaluable pocket-sized book containing memoranda and data important for every physician, and ruled blanks for recording every detail of practice. The Weekly, Monthly and 30-Patient Perpetual contain 32 pages of data and 160 pages of classified blanks. The 60-Patient Perpetual consists of 256 pages of blanks alone. Each in one wallet-shaped book, bound in flexible leather, with flap and pocket, pencil and rubber, and calendar for two years. Price by mail, postpaid, to any address, $1.25. Thumb-letter index, 25 cents extra. Descriptive circular showing the several styles sent on request. Lea & Febiger, Publishers, Philadelphia and New York.

This handsome pocketbook designed for the professional use of physicians comes to our table with all its old features of value and some new ones, making it, by and large, one of the most useful articles of the active practitioner's outfit. The description given in the heading is sufficiently ample to apprise the intending purchaser of its scope, but there are many memoranda relating to emergencies, as well as ordinary bedside practice that possess great practical utility. These relate to the examination of urine, incompatibles, treatment of asphyxia, dosage, ligation of arteries, and therapeutic hints of various kinds. It is not easy nowadays to get along without a book of this sort.

---

**Textbook of Nervous Diseases and Psychiatry** for the use of students and practitioners of medicine, by **Charles L. Dana**, A.M., M.D., LL.D., professor of nervous diseases in Cornell University; neurologist to the Montefiore Hospital; neurologist to the Woman's Hospital, etc. Seventh edition. Two hundred and sixty-one engravings; three plates in black and colors, pp. 782. New York: William Wood & Company. 1908. (Price, cloth $5.00 net.)

The several preceding editions of this admirable work have received extended notice in these columns. In this one will be found many additions, as well as adequate general revision, making it stand abreast of the science of neurology at the present day.

The finer anatomy of the nervous system, the histology of the neuron, together with the anatomy and physiology of the brain are all suitably presented. The additions in some instances have rendered the rewriting of a number of chapters necessary, while many illustrations have been added to the text. Dana is a recognised master in his chosen field and has given to the profession a book that is at once the favorite of the specialist and the student of neurology.

---

**A Manual of Diseases of the Nose and Throat.** By **Cornelius G. Coakley,** M.D., Clinical Professor of Laryngology in the University and Bellevue Hospital Medical College, New York. New (4th) editon, 12mo, 604 pages, with 126 engravings and 7 colored plates. Lea & Febiger, Philadelphia and New York, 1908. (Cloth, $2.75 net.)

We have had occasion previously to write in these columns of the quality of this manual, of its excellence we may add. It deals with almost the greatest specialty; at all events with diseases affecting the comfort, and welfare of the largest number of people. Coakley is a man of great experience and positive convictions, expressing them with emphasis and clearness. As an example of the author's teachings, for he is a clinician as well as a writer and teacher, we may point to his dissertation on the antra,—the sinuses—and their diseases, as fully sustaining our statements or comment at our hands. This edition has been revised with care, embracing every improvement of value and every method of practical worth. The chapter on therapeutics will be read with interest. Coakley is strong on examination, diagnosis, and treatment.

---

**Consumption:** How to Prevent it and How to Live With It. By **N. S. Davis,** A.M., M.D., Professor of Principles and Practice of Medicine, Northwestern University Medical School, Chicago. Second edition. 12mo. 172 pages. F. A. Davis Company, Publishers, Philadelphia. (Cloth, $1.00.)

The interest in the disease dealt with in this monograph has increased of late to such a degree, that the laity is almost as intelligent in reference to many phases of prevention as the average physician. This book was recognised at once when it first appeared in 1891, as a helpful guide for the patient and as being full of useful hints to the physician. It has now been revised to include most valuable information for everyone interested in a disease that has come now within the range of prevention and cure. The ravages of the malady have heretofore appalled those who have made a study of the subject. Thanks to such efforts as this author has put forth, we are now coming to see the light on a disease whose terrors have been reduced many fold.

**Pain.** Its Causation and Diagnostic Significance in Internal Diseases. By **Dr. Rudolph Schmidt,** Assistant in the Clinic of Hofrat von Neusser, Vienna. Translated by **Karl M. Vogel** and **Hans Kinsser,** College of Physicians and Surgeons, New York. 12mo, pp. 326. Philadelphia and London: J. B. Lippincott Co. (Cloth, $3.00).

Not since the appearance of Mr. Hilton's monograph on Rest and Pain, now some thirty years ago, has such a complete analysis of the symptomatology of pain been presented to the profession as we find in Dr. Schmidt's treatise. It is quite often difficult to read aright the significance of pain and to assign to it a proper place as a sign of disease; indeed, it often masks the real, the true diagnostic evidences, and thus may become misleading. Therefore, it becomes necessary to study it alone, apart from other manifestations of disease, as well as in conjunction with them. This author points the way to do this, the way to "correct analyses and logical deductions." Pain is the all-important symptom to the patient, and the first duty of the physician often is to relieve the distress, the suffering, before he can form a correct opinion as to the malady. In this book will be found a guide to the solution of a difficult problem, and we doubt not every clinician will welcome it to his library.

---

**Refraction of the Eye,** by **Harry C. Parker,** M.D., Clinical Professor of Ophthalmology, Indiana University School of Medicine, Indianapolis. With 106 illustrations. Philadelphia and London: W. B. Saunders Company. 1908. (Price, $1.25 net.)

The importance of proper refraction can scarcely be overestimated. The student, in particular, should be instructed with care on this subject that he may never afterward deal lightly with eyes needing correction lenses. Too often such conditions are given over to persons inadequately instructed in the principles of optics, much to the discomfiture of the sufferers. Parker in this monograph deals with the topic seriously, and, withal, scientifically and his book, in our opinion, should find itself in the hands of every junior ophthalmologist.

---

**The Cure of Rupture by Paraffin Injections** by **Charles C. Miller,** M.D. Small 12 mo, pp. 81. Published by the Author, 70 State St., Chicago. (Prepaid $1.00.)

This essay may with propriety be entitled "Hernia cured while you wait." The author seems to regard the injection of paraffin as an efficient means of curing hernia, as being perfectly safe, and as feasible for employment in the physician's office. All things appear possible in these days of rapidity of movement, but we are inclined to adhere to the old fashioned knife as the most reliable cure for this affliction. However, it will do no harm to read this primer and we commend the author as having the courage of his convictions.

**Reference Handbook for Nurses** by **Amanda K. Beck**, Graduate of the Illinois Training School for Nurses. Second edition. Revised. Philadelphia and London. W. B. Saunders Company. 1908. (Price, $1.25 net.)

Nurses need just such a book as this,—one prepared by a practical nurse, containing practical material. There is nothing in this manual that every nurse will not need sometime in her career, and when she does need it she will want it badly; hence she had better get the book now. It is of such convenient size as to find an appropriate place in the handbag, therefore can always be at hand,—a point in its favor of no mean importance. A few useful illustrations have been added to this edition, thus making the book about as complete as can be without materially increasing its size, which would be a mistake in our view.

---

**Manual of Clinical Diagnosis** by **James Campbell Todd,** Ph.B., M.D., associate professor of pathology in Denver and Gross College of Medicine, University of Denver, etc. Illustrated. W. B. Saunders Company, Philadelphia and London. 1908. (Price, $2.00 net.)

The title of this book should read "Laboratory Methods of Clinical Diagnosis." It would then not be misleading, as it deals only with those methods. The usual bedside methods are not detailed in this book. We must not be understood as offering this criticism in derogation of the material contained in the pages of this manual. On the other hand, we regard the entire text as most valuable. The author is painstaking, scientific, correct; his book is full of instructive matter, and is to be commended to every student who would perfect himself in the art of diagnosis, who would make a name for himself as a diagnostician. The beauty of the volume is striking; its make-up appeals to the lover of handsome books.

---

**The Ready-Reference Handbook of Diseases of the Skin.** By **George Thomas Jackson,** M.D., Chief of Clinic and Instructor in Dermatology, College of Physicans and Surgeons, New York. Sixth edition. 12 mo, 737 pages, with 99 engravings and 4 plates, in colors, and monochrome. Lea & Febiger, Publishers, Philadelphia and New York, 1908. (Cloth, $3.00, net.)

The profession, both general and special, the dermatologist and general practitioner, one and all are so familiar with this handbook, that little need,—indeed, little new can be said about it at the present time. It is almost a classic in the literature of dermatology, standing alone as to its value and importance among the handbooks in this specialty. This edition has been thoroughly revised, the literature of dermatology studiously examined and every important point incorporated in the text, so it may be stated as a general proposition that no dermatological work of its class could be more complete. The new articles added will increase interest in the book. They are upon black tongue, dermatitis verrucosa, keratosis follicularis contagiosa, keratosis senilis, lichen obtusus, melung, pseudopelade, and sporotrichosis hypodermica. The pathology in the book has been revised and added to—making the work as nearly complete as may be.

---

**Pulsating Exophthalmos.** By **George E. de Schweinitz,** M.D., Professor of Ophthalmology in the University of Pennsylvania, and **Thomas B. Holloway,** M.D., Instructor in Ophthalmology. Small octavo. p. 124. Philadelphia and London: W. B. Saunders Co. (Cloth, $2.00).

The subject of this monograph has not before been separately dealt with, as far as we are informed. The affection is not met with frequently, hence this brochure will be welcomed by every ophthalmologist. From 1805 to July, 1907, 313 cases of pulsating exophthalmos have been recorded. These authors have analysed sixty-nine cases in addition to those referred to, the object being to determine, if possible, those surgical procedures likely to prove of greatest value in controlling the symptoms of this disease. The cases are tabulated at the end of the book and so made available for immediate reference. It is of the higher class medical literature.

---

**Cataract Extraction.** A Series of Papers with discussions read before the Ophthalmological Section of the New York Academy of Medicine, 1907-1908. Edited by **J. Herbert Claiborne,** M.D., Instructor in Ophthalmology, Cornell University Medical College. Small octavo, pp. 169. New York: William Wood & Co. (Cloth, $2.00.)

This group of essays on the important subject of cataract extraction will prove of interest to every ophthalmologist. They are written not only by capable men but have been attractively edited and printed and will serve to adorn the library, as they could not if allowed to be buried in the journalistic chaos of the attic. Dr. Claiborne deserves the thanks of his colleagues for the part he has performed so well.

---

**International Clinics.** A Quarterly of Illustrated Clinical Lectures and especially prepared articles on Treatment, Medicine, Surgery, Neurology, Pediatrics, Obstetrics, Gynecology, Orthopedics, Pathology, Dermatology, Ophthalmology, Otology, Rhinology, Laryngology, Hygiene and other topics of interest to students and practitioners. Edited by **W. T. Longcope,** M.D., Volume II., eighteenth series. Philadelphia and London: J. B. Lippincott Co. 1908. (Cloth, $2.00.)

This volume contains twenty-four articles, distributed among the various branches of medicine as follows: five on treatment; five on medicine; four on surgery; three on gynecology; two on ophthalmology; one on dermatology; one on orthopedics; one on pediatrics; and two on pathology. Of the more notable contributions may be mentioned that of Louis Fischer on the treatment of scarlet fever, that of Behan on the changes in the outlines of the heart, etc., that by John G. Cecil on valvular disease of the heart, that by Cumston on perinephritic abscess in children, that by Sampson on the clinical manifestations of uterine cancer, that by Louis Frank on pregnancy complicated by uterine fibromata in a hemiplegic, and that by Simon on recent research into the pathology of malignant disease. All the articles, however, are of sufficient merit to justfy the statement that this number is one of unusual value. It is well illustrated by a large number of figures and plates that add very much to the quality of the volume.

---

**The Principles and Practice of Hydrotherapy.** By **Simon Baruch,** M.D., professor of hydrotherapy in Columbia University (College of Physicans and Surgeons) New York. Third edition. Octavo, pp. 544. Illustrated. New York: William Wood & Co. (Cloth, $4.00.)

Every experienced physician knows full well that water is a valuable therapeutic aid in the treatment of disease,—some diseases; every competent physician is equally well aware that water is not a cure-all,—that alone it is not adequate in many diseases. And yet it is quite possible if one should be limited, by some mischance, to one remedy, water would fill the largest therapeutic place.

To one unfamiliar with the various uses of water in medicine this book will supply the needed information. It is written on a scientific plane though, perchance, somewhat enthusiastically; yet this scarcely detracts from its merit as a therapeutic guide. This edition is constructed on the same general lines as its predecessors, some new material being added to make it conform to the author's further experience. It is the best exponent of hydrotherapy at the present time.

---

**The Physician's Visiting List** (Lindsay & Blakiston's) for 1909. Fifty-eighth year, with dose table revised in accordance with U. S. Pharmacopeia, 1905. Philadelphia: P. Blakiston's Son & Company, 1012 Walnut Street.

Blakiston's visiting list for physicians for the year 1909, is in the field with promptitude. This is the fifty-eighth year of its publication, with all its old valuable features retained and such new ones added as are necessary to keep it abreast of existing requirements. It contains among other things calendars for 1909, and 1910; a table for calculating the period of gestation; a dissertation on incompatibility; the treatment of poisoning; metric system of weights and measures; table converting apothecaries weights and measures into grams; dose table, both English and metric systems; asphyxia and apnea treatment; comparison of thermometers; these, beside the weekly pages for recording professional work, cash account, obstetric and vaccination engagements and space for other valuable material. It is compact, ample for all purposes, and is published in three forms,—regular edition, perpetual edition, and monthly edition.

**The Natural History of Cancer,** with special reference to its Causation and Prevention. By **W. Roger Williams,** Fellow of the Royal College of Surgeons. Octavo, pp. 519. Illustrated. New York: William Wood & Co. 1908. (Cloth, $5.00.)

The literature of cancer is now extensive and constantly increasing in volume if not in quality. It is a disease that is attracting the attention of those students who would ascertain its cause, and is being investigated from all viewpoints, some seeking to prove its parasitic origin, while others are content to establish its starting point if possible, without regard to theoretic cause. Williams presents facts that he has adduced in the study of the natural history of the disease, massed with great care, and which cannot fail to aid in the further study of cancer from any and all viewpoints.

Many of his statements are surprising if not startling. This author, for example, says he has never found cancer among prostitutes, and that persons of drunken or dissolute habits are seldom afflicted with the disease. He gives statistics bearing on the topographical frequency of cancer that are interesting if not instructive. Williams is a logical observer and does not rush into print with a theory to establish; but, on the other hand, groups his facts with care and presents them duly analysed for the inspection of those interested. His book represents enormous labor and helps to bring light into a dark field. We doubt not it will prove of invaluable aid in the solution of the most difficult problem undertaken by modern investigators.

---

**Subcutaneous Hydrocarbon Protheses.** By **F. Strange Kolle,** M.D., Author of the "Recent Roentgen Discovery," etc. 12 mo, pp. 153. New York: The Grafton Press. (Cloth, $2.50.)

Under another name this author describes the method of subcutaneous injection of paraffin for correction of facial deformities. The subject is presented with skill and whatever of value there may be in the method is set forth attractively. The printing is superb and the little brochure will prove an addition to the library of the rhinologist who may wish to preserve all the novelties of his art. We may add that this is the best brochure on this topic we have yet seen.

---

**Insomnia and Nerve Strain.** By **Henry S. Upson,** M.D., professor of diseases of the nervous system in the Western Reserve University, Cleveland, O. 12 mo, pp. 142. Illustrated. New York and London: G. P. Putnam's Sons. 1908.

This essay affords most interesting reading to those who would delve into the mysterious realm of mental hebetude, of the psychoses that cause unrest, even, in some instances, actual insanity. Insomnia is generally due to physical fault or faults, and this writer tells of this fact and how to correct such conditions, thus bringing about relief. All mental aberrations attract attention because of pity or fear, and create apprehension in the minds of relatives or friends. To interpret the symptoms aright may not be easy to the inexperienced, but this monograph furnishes a helpful guide to a solution of many conditions otherwise obscure or unfathomable. It is quite as important for the general practitioner to possess it as that the specialist should place it among his choice collection. It will prove useful to both the alienist and the family doctor.

